# Personality Differences of Brain Networks in Placebo Analgesia and Nocebo Hyperalgesia: A Psychophysiological Interaction (PPI) Approach in fMRI

**DOI:** 10.1155/2020/8820443

**Published:** 2020-10-19

**Authors:** Yu Shi, Shimin Huang, Hongrui Zhan, Yaping Wang, Yanyan Zeng, Guiyuan Cai, Jianming Yang, Wen Wu

**Affiliations:** ^1^Department of Rehabilitation, Zhujiang Hospital, Southern Medical University, Guangzhou 510282, China; ^2^Department of Physical Medicine and Rehabilitation, The Fifth Affiliated Hospital of Sun Yat-sen University, Zhuhai 519000, China; ^3^Department of Radiology, Zhujiang Hospital, Southern Medical University, Guangzhou 510282, China

## Abstract

It is generally believed that the placebo response can elicit an analgesic effect, whilst the nocebo response can elicit a hyperalgesia effect in pain. Placebo analgesia and nocebo hyperalgesia effects are increasing concerns for researchers. Growing evidence suggests personality differences have an impact on both placebo and nocebo effects. However, previous studies have not reached a unified conclusion. We designed this study to explore the personality differences of functional magnetic resonance imaging (fMRI) signals in placebo response and nocebo response by using psychophysiological interaction (PPI) analysis. 30 healthy subjects underwent conditioning induction training to establish expectations of placebo effect and nocebo effect, and then, all subjects completed the following experimental procedures: (1) baseline scanning, (2) acute pain model establishment, (3) pain status scanning, and (4) pseudorandom scanning of block design of placebo response or nocebo response. Behavioral data were collected after each scan. The results of this study showed that (1) there were significant differences of VAS placebo intervention between the extrovert group and the introvert group (*p* = 0.004); (2) there were significant differences of VAS nocebo intervention between the extrovert group and the introvert group (*p* = 0.011); (3) there were significant differences between the VAS placebo intervention and VAS pain status (baseline) in both the extrovert group (*p* < 0.001) and the introvert group (*p* = 0.001); (4) there were significant differences between the VAS nocebo intervention and VAS pain status (baseline) in both the extrovert group (*p* = 0.008) and the introvert group (*p* < 0.001). Moreover, there were significant differences in the brain network for placebo and nocebo responses between different personalities. We found that (1) deactivation differences of the pain-related network and limbic system play an important role in personality differences associated with placebo analgesia and (2) differences of control of anxiety and activation of dorsolateral prefrontal cortex may cause the personality differences observed in nocebo hyperalgesia.

## 1. Introduction

As an important psychological response, the placebo effect and nocebo effect have attracted increasing attention from researchers [1, 2]. The terms placebo response and nocebo response refer to positive and negative cognitive, emotional, and behavioral modulation of outcome [3, 4]. In pain research, researchers found that if the subjects had a good expectation of an analgesic effect (placebo effect), there would be a better analgesic effect [5–7], whilst the nocebo effect is defined as the heightened pain response to a low-pain inducing and/or innocuous stimulus purported to increase pain or unpleasant symptoms [8].

Previous reports on placebo as well as nocebo effects have focused on neurotransmitters and nerve signal transduction pathways. Some scholars have suggested that the placebo analgesia effect is based on the opioid-mediated analgesia system (OMAS) [9–11], whilst the nocebo hyperalgesia effect is mediated by the cholecystokininergic system (CCK system) [12] and hypothalamic-pituitary-adrenal axis (HPA) hyperactivity [13]. However, it is noteworthy that the above studies cannot fully study the placebo effect and nocebo effect brain network, which is based on the advanced neurological function of the brain [14].

Brain imaging technology has emanated a pivotal approach to study the brain's advanced nervous activities and can enable a comprehensive macro analysis of the brain network [15, 16]. Consequently, many researchers have begun to use brain-imaging technology to study the brain's responses to placebo analgesia and nocebo hyperalgesia [17]. Some researchers have suggested that the placebo effect could activate the anterior cingulate cortex (ACC), amygdala (AMYG), prefrontal cortex (PFC), insula (IC), and thalamus (THS) and that the activation of these areas is closely related to the placebo-mediated analgesic effect [18–20]. Furthermore, Schmid et al. [21] suggested that the brain areas of the THS, IC, AMYG, and midcingulate cortex (MCC) are closely related to the effect of nocebo hyperalgesia.

At present, there are many hypotheses about the relationship between placebo and nocebo effects. Some studies believe that the brain networks of placebo effect and nocebo effect are two relatively independent networks. Bingel et al. found that the nocebo response would significantly activate the hippocampus (HP), but no activation of the HP was found in the placebo response, when they studied the effect of anticipation on the analgesic effect of fentanyl [12, 22]. However, some studies have shown that the placebo effect and the nocebo effect may be controlled by the same brain areas, and the difference between them is only due to the different activation status of the relevant brain areas [23]. Therefore, the brain network mechanisms of placebo and nocebo effects remain unclear.

Some studies have found that extroverts are more likely to be encouraged by speech in pain treatment than introverts, resulting in an obvious placebo effect and a better analgesic effect. Some scholars have confirmed that extroverts have higher prefrontal activity and are more likely to induce a reward effect of the dopamine system [24]. This indicates that there may be differences in neural networks among different personality trait groups, and different personality trait groups have different brain network activities. Based on the above studies, we hypothesize that there are significant differences in the placebo effect and nocebo effect of brain networks of introverts and extroverts, which can help to reveal the brain mechanisms of these effects.

To effectively study the effects of the brain network mechanisms of placebo analgesia and nocebo hyperalgesia, we need to examine their respective independent brain network responses, not only separately but also in terms of the associations between them. To date, in the field of brain imaging, only a few papers have studied personality differences in the placebo effect. Furthermore, none have, as yet, investigated personality differences in nocebo hyperalgesia nor directly compared personality differences in placebo and nocebo effects. Therefore, a more in-depth study is necessary.

Moreover, it is worth cognizing that a large number of previous researches focused on the placebo analgesia as well as nocebo hyperalgesia effects of chronic pain [25]. It is widely accepted that chronic pain can alter the structure of the brain as a result of the long-term course of disease [26], thereby affecting the brain network's response to the effects of placebo analgesia, as well as nocebo hyperalgesia. Establishing acute pain models in healthy subjects can, therefore, effectively overcome this potentially confounding problem. Furthermore, few previous studies have used the acute pain model to study nocebo hyperalgesia.

As an important structure, the ACC serves a pivotal role in the higher nervous functions of the brain. In the pain-related network, the ACC is mainly involved in the emotional motivation and cognitive attention of pain. Some subareas of the ACC may also participate in the identification of pain perception components [27]. The ACC is widely associated with the IC and the primary somatosensory cortex area (S1) and is responsible for processing pain signals from the IC [28]. The ACC is also an important structure in the reward and dopamine systems and plays a core function in the placebo response [29]. As an important part of the ACC, rostral ACC (rACC) undertakes most of the above functions and plays an important role in emotion regulation [30], analgesic control [31], and avoidance behavior [32]. Hence, the rACC option as a region of interest (ROI) is a vital approach of exploring one of the core nodes of the brain's network of placebo analgesia, as well as nocebo hyperalgesia.

In the present experiment, we used a mature acute lower back pain (ALBP) model [33] to explore personality differences in the brain's response in placebo analgesia and nocebo hyperalgesia. We manipulated subjects' expectations of the performance of two patches, with one patch labeled “analgesic patch” (positive expectancy) and another one “algetic patch” (negative expectancy). We then inspected the pain scales and the variations in the fMRI signal before and after the different “treatments.” Furthermore, a psychophysiological interaction (PPI) method was designed to investigate the effective connectivity of placebo analgesia and nocebo hyperalgesia. With this report, we hope to further our comprehension of personality differences in placebo analgesia and nocebo hyperalgesia and offer a foundation for future researches.

## 2. Methods

### 2.1. Participants

Herein, we recruited participants by posting in Zhujiang Hospital. The participants lived in almost identical areas, and all were right handed. The inclusion criteria constitute (1) never having taken part in a psychological experiment, (2) a body mass index in the range of standard ± 10%, (3) no psychiatric or medical conditions (consisting of multiple sclerosis, epilepsy), (4) no pain (constituting dysmenorrhea) or drug (i.e., antipyretics and sleeping pills) experienced in the last month, and (5) self-rating anxiety scale (SAS) and self-rating depression scale (SDS) scores < 50 (lower than 50 indicates “normal”). The general exclusion maxims constituted organic brain disease, a history of skull or brain damage, substance dependency, severe neurological illnesses, metallic constituents in the body, claustrophobia, and use of analgesic drugs (within the last month). The Ethics Committee of Zhujiang Hospital ratified all the experiments, as well as protocols [34]. We provided all the participants with a choice of exiting the study and removing their data whenever there were issues on the methodological requirement for deception in the experimental ideology. No participant raised any issue, and all gave consent for their data to be utilized.

The Eysenck Personality Questionnaire (EPQ) [35, 36] was used to distinguish introverts from extroverts. The internal consistencies of the EPQ were evaluated from four dimensions: N = Neuroticism, E = Extraversion, P = Psychoticism, and L = Lie/Social Desirability. The results of reliability analysis of the EPQ showed that the scale is applicable to the subjects (N: *α* = 0.83, E: *α* = 0.80, P: *α* = 0.62, and L: *α* = 0.78). Introverts were placed in the introvert group (IG), and extroverts were placed in the extrovert group (EG).

### 2.2. Experimental Procedures

**Figure 1:**
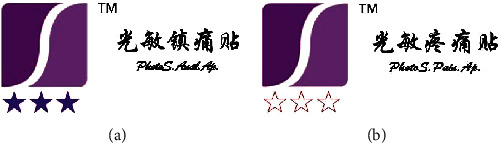
Design sketch of two patches: (a) one for the “photosensitive analgesic patch” and (b) one for the “photosensitive algetic patch”.

In this study, we designed two patches to convey psychological suggestions: one was labeled “photosensitive analgesic patch” (positive expectancy), and one was labeled “photosensitive algetic patch” (negative expectancy). The two patches were very similar in appearance to the analgesic patch commonly used in clinical practice (see Figure 1).

**Figure 2:**
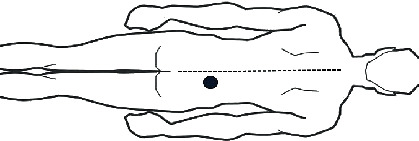
ALBP model location.

To obtain a stable state of acute pain, we used the ALBP model. The ALBP model applied was based on our previous study [33]. In accordance with this model, the point of injection was located 2 cm tangential of the spinous process of the fourth lumbar vertebra. After that, filling of an indwelling needle (24 gauge) was accomplished with sterile hypertonic saline (10 mL, 5%); then it was attached through a long linking tube to a computer-controlled power injector (Spectris Solaris EP; Medrad, Inc., Warrendale, PA, USA) prior to its vertical insertion into the above-documented area at a depth of 1.5 cm. After one minute, the ALBP subject received the hypertonic saline via intramuscular injection. This injection constituted a bolus injection (0.1 mL within 5 s) and ensuing continuous injections (0.15 mL/min) to generate constant ALBP [33] (see Figure 2).

#### 2.2.1. Training Session

In this session, we acquainted the participants with the ALBP model as well as the visual analogue scales (VAS) that they would be using to rate their pain. We inspected the intensity of the pain on a 10 cm VAS anchored with “no pain” (0) and “worst pain imaginable” (10). Moreover, we inspected the pain unpleasantness (i.e., distressing, as well as horrible) using a 10 cm local mood scale anchored with “infinitely small” (0) and “excruciating” (10). At the same time, we also recorded any feelings of discomfort experienced by the subjects to prevent the occurrence of any adverse reactions. Behavioral tests were conducted at the end of the experiment, and the subjects reported changes in pain scores which were caused by the interventions.

#### 2.2.2. Behavioral Conditioning Session

We enlightened all the participants that the purpose of the study was to inspect the analgesic effects of the photosensitive analgesic patch and the algetic effects of the photosensitive algetic patch on their experience of pain. In order to make the subjects adapt to the experimental environment better, the process was carried out in the MRI room. We told subjects that we would apply one of the two patches (the photosensitive analgesic patch or the photosensitive algetic patch) to their foot when they experienced any ALBP. We also told subjects that both patches were controlled by a laser. Patches will work when the laser irradiates and will not work when the laser does not irradiate; thereafter, the subject should feel the pain change depending on the patch applied. Both patches need to feel, and the order in which the patch was applied was random.

After the state of ALBP was stable, we effected the experimental operation. In this conditioning paradigm, we informed subjects that they would experience a change in pain depending on whether the laser was irradiated on the photosensitive analgesic patch or the photosensitive algetic patch. During the process, we informed the participants to gaze on captions on a screen. When the laser was irradiated on the photosensitive analgesic patch, the following caption was displayed on the screen: “Please experience the analgesic effect of the analgesic patch.” When the laser was irradiated on the photosensitive algetic patch, the following caption was displayed: “Please experience the algetic effect of the algetic patch.” When the laser was not irradiated on the patches, the following caption was displayed: “Patch is not working.” In this experiment, the time of laser irradiation was designed by block and the photosensitive analgesic patch or the photosensitive algetic patch run constituted a block design with six 30 s blocks of rest time (OFF block) interspersed between six 30 s blocks of stimulation (ON block). The laser was irradiated on the photosensitive analgesic patch or the photosensitive algetic patch during the ON block. After each stimulus had been administered, we displayed the VAS on the screen and subjects then recorded their pain score, which was caused by the interventions.

In reality, we reduced the rate at which the hypertonic saline was administered when the laser was irradiated on the photosensitive analgesic patch and increased it when the laser was irradiated on the photosensitive algetic patch. Only participants who could delimit the pre- and postintervention analgesic effects of the analgesic patch and algetic effects of the algetic patch were authorized to continue to the next session. That is, at least one-point lower VAS score in the analgesic patch intervention and at least one-point higher VAS score in the algetic patch intervention were considered to be able to clearly distinguish between analgesic and algetic effects.

#### 2.2.3. Scan Session

The experiment was performed in the Department of Radiology of Zhujiang Hospital. We obtained the structural as well as functional scans via 3.0 T Philips Achieva MRI System (Royal Philips Electronics, Eindhoven, The Netherlands) equipped with an 8-channel head array coil for the echo planar imaging. Consequently, the images were axial as well as parallel to the anterior commissure-posterior commissure line, covering the entire brain. We collected the structural images before accomplishing functional imaging via a T1-weighted fast spin echo sequence (repetition time/echo time = 25/3 ms, flip angle = 30°, matrix = 256 × 256, thickness = 5 mm, slice = 24, and slice gap = 0.7 mm). We conducted blood oxygenation level-dependent functional imaging via a T2∗-weighted, single-shot, gradient-recalled echo planar imaging sequence (repetition time/echo time = 2000/35 ms, flip angle = 90°, matrix = 64 × 64, thickness = 5 mm, slice = 24, slice gap = 0.7 mm, NSA = 1, and 180 time points for an overall 360 seconds). Moreover, fMRI image acquisition was heralded by 5 dummy scans to diminish gradient distortion.

We informed subjects that the procedure for the *Scan Session* would be identical to that of the previous session (the *Behavioral Conditioning Session*). In reality, the Scan Session was developed to inspect the placebo as well as nocebo effects instigated by the expectancy manipulation in the *Behavioral Conditioning Session*. Therefore, the *Scan Session* process was comparable to that of the *Behavioral Conditioning Session*, but without manipulation of the rate at which the hypertonic saline was administered.

**Figure 3:**
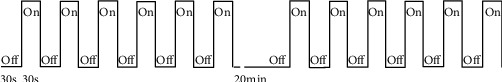
Every functional scan run for 6 min constituting six OFF–ON blocks; the duration interval between the two functional scans was 20 min. During the six ON blocks of each functional scan, the laser was irradiated on the photosensitive analgesic patch or the photosensitive algetic patch.

First, we acquired the anatomical scans of the brain prior to the fMRI scans. Incipiently, we put the participants through a baseline (normal) resting state- (rs-) fMRI scan for six minutes. An exploratory ALBP model was triggered in the right lower back muscle of each participant, as above. During the first six minutes of the ALBP state, we effected an rs-fMRI scan to inspect participants' pain sate. Following the pain rs-fMRI scan, we acquired 2 functional scans for every ALBP participant: scan 1 during photosensitive analgesic patch induction and another scan in the photosensitive algetic patch induction, in a pseudorandom approach, with ALBP happening continuously through the process of scanning. The photosensitive analgesic patch or the photosensitive algetic patch run constituted a block design of six 30 s blocks of rest time (OFF block) interjected between six 30 s blocks of inducement (ON block). The laser was irradiated on the photosensitive analgesic patch or the photosensitive algetic patch during the six ON blocks of every functional scan. Every functional scan took 6 min, and the duration interval between the two functional scans was set at 20 min to optimize washout of the sustained influences triggered by the previous intervention (see Figure 3).

Pre- and postintervention variations in subjective VAS as well as the fMRI signal induced by identical postintervention moderate pain stimuli served as the primary outcomes herein.

**Figure 4:**
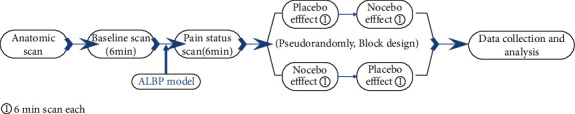
The experimental paradigm (Scan Session) for the subjects.

During scanning, subjects were instructed to focus on the captions on the screen. When the laser was irradiated on the photosensitive analgesic patch, the following caption was displayed on the screen: “Please experience the analgesic effect of the analgesic patch-30 s”; when the laser was irradiated on the photosensitive algetic patch, the following caption was displayed: “Please experience the algetic effect of the algetic patch-30 s”; and when the laser was not irradiated on the patches, the following caption was displayed: “Patch is not working-30 s.” After administering each induction, we broadcasted the VAS on the screen and subjects recorded their pain scores, which were caused by the interventions (see Figure 4).

### 2.3. Preprocessing of Functional MRI Data

We preprocessed the fMRI image and employed the Data Processing & Analysis for Brain Imaging (DPABI, http://rfmri.org/dpabi) tool by routines in MATLAB R2013b to inspect it. The blood oxygen level-dependent (BOLD) time series preprocessing stages constituted the slice-time correction, motion correction, normalization to the Montreal Neurological Institute (MNI) templates, spatial smoothing, and finally temporal band-pass filtering. We employed the motion time courses to accomplish selection of the participant's head movements of <2 mm in translation and 2° in rotation that we utilized for subsequent analysis (no participants were excluded). We utilized the symmetric echo planar imaging templates to normalize the functional images of every participant and resampling accomplished at a resolution of 3 mm × 3 mm × 3 mm. We employed a 6 mm full width at half-maximum (FWHM) Gaussian kernel to spatially smooth the normalized functional images. Moreover, we performed voxel-wise linear trend removal as well as temporal band-pass filtering (0.01–0.08 Hz) to diminish the impacts of very low-frequency drift as well as high-frequency noise.

### 2.4. Definition of Seed Region

**Figure 5:**
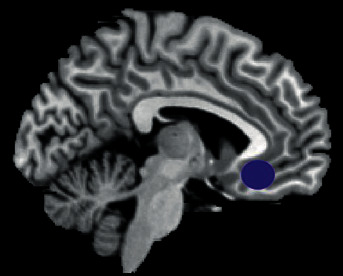
The location of ROI (rACC).

To be on the same side as the intramuscular part, the data determination of left side of rACC for the ROI (3 × 3 × 3 mm^3^) was centered on the findings of a previous MRI research [37]. MNI brain region coordinates were chosen as the central voxel ROI (*x* = −5, *y* = 25, *z* = −10) (see Figure 5).

### 2.5. Psychophysiological Interaction (PPI) Analysis

Psychophysiological interaction (PPI) assessment is a hypothesis-propelled strategy to inspect context-distinct variations in effective connectivity (EC) between one or more *a priori* defined brain regions of interest, as well as the rest of the brain. PPI evaluation simply documents which voxels throughout the entire brain escalate their signal alterations linked to the seed ROI during, as well as regulated by task implementation. PPI examination constitutes a simple brain connectivity approach that delineates the activity in one brain region by the crosstalk between another region's activity and a psychological factor and an interregional correlation analysis [38]. This is attained via comparing the connectivity in one context (in this case, “placebo effect or nocebo effect-ON status”) with another context (here, “pain status-OFF status”).

Employing the PPI evaluation methodology effected in SPM12, we inspected if the rACC was differentially linked to other brain regions with regard to each other, as well as to the experimental context of placebo status (or nocebo status) versus pain status. Firstly, we modeled the preprocessed task functional data using a general linear model (GLM). Two regressors of GLM constituting the inducement task ON status, as well as the OFF status, were modeled via a boxcar function that convolved with the canonical hemodynamic response function in SPM12 (using a 128 s high-pass filter). Secondly, for each subject, we extracted an average BOLD signal time course from the seed region, defined as a 3 mm sphere around coordinates derived from previous reports. The rACC inspection was centralized at MNI coordinates -5, 25, and -10. The psychophysiological interaction term (PPI regressor) was described as the cross-product of the physiological activity and psychological variable. Hence, a GLM was created using the psychophysiological interaction term, the physiological activity, and the psychological variable as the regressors. Each PPI examination was accomplished individually for each participant and the seed region, focusing on two complementary contrasts, namely, “placebo (or nocebo)-ON status” greater than “pain-OFF status” and “pain-OFF status” greater than “placebo (or nocebo)-ON status.” Group comparison: the consequential contrast images were entered second level within and between-group evaluations, using one- and two-sample *t*-tests, respectively.

### 2.6. Statistical Analysis

We utilized the SPSS 20.0 software (SPSS, Chicago, IL, USA) to inspect the descriptive statistics (mean ± SD) for VAS and other data. Two-sample *t*-tests were used for intergroup comparison, and paired *t*-tests were used for intragroup comparison. All statistical assessments were two tailed. *p* < 0.05 signified statistical significance, consistent with the preliminary status of the trial.

We used the false discovery rate (FDR) with a significance level of *p* < 0.05 and cluster size of 30 or greater at the voxel level to correct the PPI results for multiple comparisons.

## 3. Result

30 healthy adults (13 introverts) completed the study. In the IG, there were statistical differences between placebo status and pain status, as well as between nocebo status and pain status (*p* < 0.05; see Table 1). In the EG, there were also statistical differences between placebo status and pain status and between nocebo status and pain status (*p* < 0.05; see Table 1). Moreover, there were statistical differences of VAS between IG and EG, in both placebo status and nocebo status (*p* < 0.05; see Table 1). However, there was no statistical difference in the VAS scores for pain status (*p* > 0.05; see Table 1). And there was no statistical difference in the SAS scores or SDS scores between IG and EG (*p* > 0.05; see Table 1).

### 3.1. PPI Analysis Results

#### 3.1.1. Brain Response of Placebo Effect in IG

**Figure 6:**
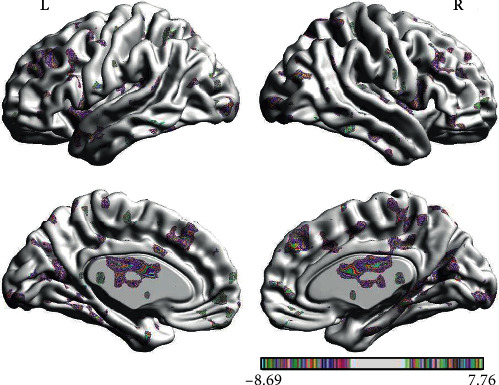
Regions showing significantly different EC with the rACC in placebo status compared with pain status (IG).

In the PPI map of the rACC, the results showed that compared with pain status (OFF block), placebo status (ON block) had decreased EC with the cerebellum posterior lobe (CPL), parahippocampal gyrus (PHP), IC, THS, dorsolateral prefrontal cortex (DLPFC), posterior cingulate cortex (PCC), and supplementary motor area (SMA) and increased EC with fusiform gyrus (FG) (see Table 2 and Figure 6).

#### 3.1.2. Brain Response of Placebo Effect in EG

**Figure 7:**
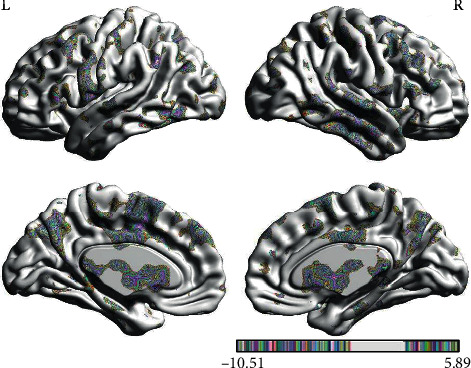
Regions showing significantly different EC with the rACC in placebo status compared with pain status (EG).

In the PPI map of the rACC, the results showed that compared with pain status (OFF block), placebo status (ON block) had decreased EC with AMYG, middle temporal lobe (MTL), CPL, lingual gyrus (LG), HP, inferior temporal lobe (ITL), orbitofrontal cortex (OFC), angular gyrus (AG), pregenual anterior cingulate cortex (pgACC), secondary somatosensory area (S2), SMA, DLPFC, ventromedial prefrontal cortex (VMPFC), and precuneus and increased EC with brainstem (BRS) (see Table 3 and Figure 7).

#### 3.1.3. Brain Response of Placebo Effect in IG vs. EG

**Figure 8:**
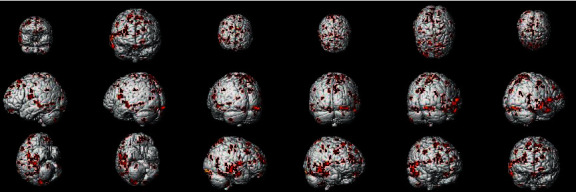
Regions showing significantly different EC with the rACC in placebo status (IG) compared with placebo status (EG).

In the PPI map of the rACC, the results showed that in placebo intervention, IG had increased EC in the HP, rolandic operculum (RO), IC, PCC, MCC, and SMA when compared with EG. In addition, the rACC showed significantly decreased EC with cerebellum anterior lobe (CAL) in IG as compared to EG (see Table 4 and Figure 8).

#### 3.1.4. Brain Response of Nocebo Effect in IG

**Figure 9:**
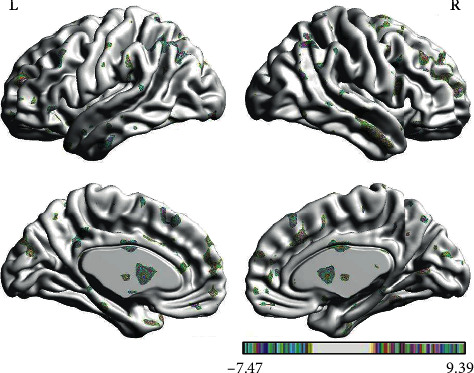
Regions showing significantly different EC with the rACC in nocebo status compared with pain status (IG).

In the PPI map of the rACC, the results showed that compared with pain status (OFF block), nocebo status (ON block) had increased EC with CPL and decreased EC with BRS, ITL, OFC, DLPFC, and S1 (see Table 5 and Figure 9).

#### 3.1.5. Brain Response of Nocebo Effect in EG

**Figure 10:**
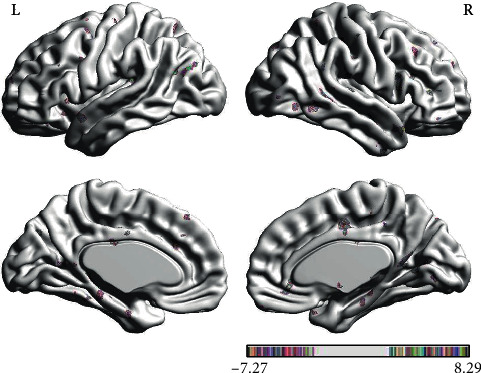
Regions showing significantly different EC with the rACC in nocebo status compared with pain status (EG).

In the PPI map of the rACC, the results showed that compared with pain status (OFF block), nocebo status (ON block) had increased EC with BRS, caudate (CAU), and IC (see Table 6 and Figure 10).

#### 3.1.6. Brain Response of Nocebo Effect in IG vs. EG

**Figure 11:**
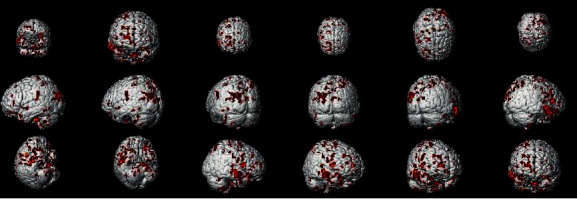
Regions showing significantly different EC with the rACC in nocebo status (IG) compared with nocebo status (EG).

In the PPI map of the rACC, the results showed that in nocebo intervention, IG had increased EC in the BRS, ITL, OFC, and DLPFC, when compared with EG. In addition, the rACC showed significantly decreased EC with CPL, MTL, and IC in IG as compared to EG (see Table 7 and Figure 11).

#### 3.1.7. Brain Response of Placebo vs. Nocebo in IG

**Figure 12:**
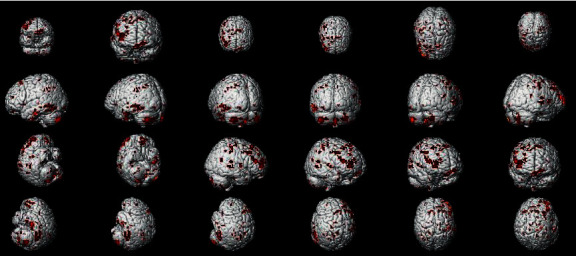
Regions showing significantly different EC with the rACC in placebo response compared with nocebo response (IG).

In the PPI map of the rACC, the results showed that compared with nocebo response, placebo response had increased EC with FG and HP and decreased EC with CPL, BRS, THS, PHP, MCC, IC, and DLPFC in IG (see Table 8 and Figure 12).

#### 3.1.8. Brain Response of Placebo vs. Nocebo in EG

**Figure 13:**
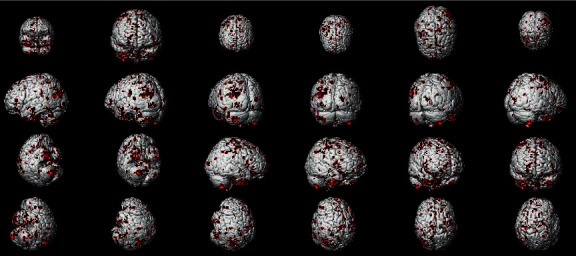
Regions showing significantly different EC with the rACC in placebo response compared with nocebo response (EG).

In the PPI map of the rACC, the results showed that compared with nocebo response, placebo response had decreased EC with CPL, THS, RO, CAU, PHP, pgACC, MCC, MTL, MFC, and SMA in EG (see Table 9 and Figure 13).

## 4. Discussion

Placebo and nocebo effects are frequently seen in both clinical practice and scientific research. Our study is the first to investigate personality differences in the nocebo effect in an ALBP model and the first to examine personality differences in placebo and nocebo effects in the same group of participants.

Herein, we found statistical differences in subjective VAS scores between IG and EG. Moreover, there were remarkable differences between the two groups in terms of brain networks, revealing that the two groups of subjects had different brain network characteristics.

### 4.1. Personality Differences in Neural Modulation during Placebo Analgesia

In this study, we found that the EG had a widely decreased EC map when compared with the IG, confirming the conclusion that extroverts are more likely to have a placebo effect. In the EG, the placebo status exhibited a decreased EC with HP, AG, S2, SMA, and DLPFC. And in IG, the placebo status exhibited a decreased EC with IC, THS, DLPFC, PCC, and SMA. By definition, EC means that one nervous system acts on another nervous system at the synapse or group level [39]. In the PPI model, it reflects the activity of brain areas based on the interaction of task and time series. If the seed can affect the activity of another brain area after counting the main effects of the task and time series, this suggests a task-related connectivity between the two brain areas [40]. The EC of PPI suggests the mutual influence between the two brain areas, and there is neuromodulation from one brain area to the other. The brain areas activated in the pain networks are described as the pain-related network, and the specific areas of interest are the ACC, S1, S2, IC, THS, and PFC [41, 42]. Here, we disclosed that under the influence of placebo analgesia, the pain-related network reduces the transmission and processing of pain information as well as the sensitivity of each brain area in the matrix to sensory information in both two groups. As an important brain area and recognized sensory component, the DLPFC is implicated in emotion regulation and pain modulation [43, 44]. The decreased EC of the placebo on the DLPFC also affects its function in the pain-related network. In EG, the activity of the pain-related network was lower than that in IG, reflecting the characteristics of personality differences of the placebo effect.

It is worth noting that ACC had a decreased EC with both sides of PHP and THS. The structures as well as crosstalking regions of the limbic system participate in motivation, emotion, learning, and memory [45]. The decreased EC between ACC and PHP/THS, which would decrease the function of the limbic system, reduced the speed of negative emotional transmission and pain sensation transmission. Moreover, some studies suggested that placebo response leads to lower pain perception, whereas nocebo response leads to higher pain perception [20]. As an important functional component of pain perception, the reduction EC of ACC affects the efficiency of pain perception. This is consistent with previous conclusions [20]. As an important sensory transmission center, the IC receives information, for example, pain, itching, and sensual touch, from the ventromedial nucleus of the THS and then passes it to the ACC for sensory information processing [28]. The IC could show its ability in interoception as well as multimodal sensory consolidation in the context of pain and also serve a pivotal role in pain-linked decision-making and emotional awareness [46]. Hence, the decreased EC between the ACC and IC in the placebo analgesia in IG may reflect the potential to inhibit painful afferent sensory information and diminish pain-linked emotional, as well as cognitive processing at the cortical level. In EG, ACC had a decreased EC with DLPFC, VMPFC, and OFC. The VMPFC is a significant portion of the PFC and serves a core function in modulating and repressing our emotional reactions [47, 48]. It collects information from the vicinal environment and signals from other brain portions of the frontal lobe and then transmits emotional information to other brain areas constituting the AMYG, ACC, thalamus, and HP. The decreased EC of the VMPFC to the ACC denotes that, in placebo effect of the EG, the VMPFC decreased negative emotional signals, hence affecting the pain progression.

As an important nerve center of emotion control, the OFC plays a role in emotion processing [49, 50]. The effects of anxiety on placebo analgesia and nocebo pain have been well known; it can enhance the nocebo effect but also reduce the placebo effect [51, 52]. Pain sensation produces a lot of negative emotions, which include anxiety [53]. Our results showed that the EC between the ACC and OFC decreased in IG, which suggested that the brain network of IG would lose some control of emotion, leading to a spread of the anxiety. This may explain why the placebo network is not significant in IG.

In EG, ACC also had a decreased EC with HP and SMA. The HP is closely related to approach-avoidance conflict, which occurs when a situation is presented that can either be rewarding or punishing, meaning the ensuing decision-making is associated with anxiety [54]. As mentioned above, anxiety is considered to be an important cause of the nocebo effect, which could reduce the placebo effect [55]. The nervous center causing anxiety is related to the ACC, AMYG, HP, and BRS. The negative regulation of the HP could reduce the incidence of anxiety. At the same time, overactivation of the ACC is also considered to be an important cause of anxiety [56, 57]. The negative regulation circuit from the HP to the ACC can inhibit anxiety and reduce its likelihood of occurring, which could play a role in the placebo effect. Some studies have shown that the release of opioid systems is closely related to the activation of ACC and BRS [11, 58]. In EG, there was an increased EC between ACC and BRS, which validates the above conclusions.

### 4.2. Personality Differences in Neural Modulation during Nocebo Hyperalgesia

In this study, we found that the IG had a widely increased EC map when compared with the EG, which suggests that IG are more likely to have a nocebo effect. In IG, ACC had an increased EC with DLPFC, OFC, IC, and S1. In EG, ACC had an increased EC with CAU and IC. In nocebo status, we found that the pain-related network had widely increased EC in both IG and EG. This is consistent with heightened pain sensation. Unlike the placebo effect, the brain increases the transmission and analysis of pain information under the nocebo effect, causing the brain to produce more pain sensations [59, 60]. The positive modulation of the IC on the ACC implies that the nocebo effect would improve the sensory information transfer function of the IC and at the same time improve the processing speed of the ACC of sensory information [61], thus leading to the hyperalgesia effect. The positive regulation of the nocebo effect on the DLPFC also improves its function in the pain-related network. In addition, the ACC had an increased EC with BRS in both two groups. As an important brain area of the opioid system, activation of BRS suggests that the opioid system may also participate in the brain network response of nocebo effect, but the specific mechanism still needs further exploration. Studies have shown that under placebo analgesia, the DLPFC would send the inhibitory signal to influence the painful sensory signaling downstream-transmission pathway [10]. Our results showed that ACC had an increased EC with bilateral DLPFCs, suggesting that under the effect of nocebo response, DLPFC reverses the state of the placebo response, enhancing the transmission of information to the painful sensory signaling downstream-transmission pathway. However, this change did not occur in the EG, which may explain why the placebo effect of the IG is more obvious.

During expectations of the nocebo effect, participants are inclined to feel anxious. Reports have shown a strong correlation between the nocebo effect and anxiety induced by the CCK [62, 63]. Behavioral researches have documented the prominent function of CCK in nocebo hyperalgesia through anticipatory anxiety mechanisms [64, 65]. The activity of the IC, ACC, and DLPFC has also been frequently linked to the processing of anxiety correlated with anticipating nociceptive stimuli. Unlike the negative regulation of the placebo effect, our results show that the nocebo effect positively and significantly regulates the IC, ACC, and DLPFC, which would replicate the role of anxiety during the nocebo effect. As an important brain area for sensory conduction, the CAU plays a role in the nocebo response [66]. Our results showed that the ACC had an increased EC with CAU, suggesting that the nocebo will increase the information transfer to the ACC through the CAU.

## 5. Limitation

Whereas the results presented here clearly show personality differences in placebo analgesia and nocebo hyperalgesia, we should note two important limitations of the study. First, in this experiment, task fMRI alone was too monotonous. A combination of resting-state fMRI and event-related fMRI could make the results more abundant. Second, the fact that only young people were selected for the study, and thus differences between subjects of different ages were not examined, is also a limitation. Third, the block design in this study was a fixed model, i.e., ON block and OFF block appeared alternately, which may strengthen and remind the expectations of the subjects, and the randomized block design should be a better design.

## 6. Conclusions

To our knowledge, this constitutes the first research to examine PPI differences in the nocebo effect in an ALBP model and the first to investigate personality differences in the placebo effect and the nocebo effect in the same group of subjects. We found that the deactivation differences of the pain-related network and limbic system play an important role in personality differences associated with placebo analgesia, and differences in the control of anxiety and activation of dorsolateral prefrontal cortex may cause the personality differences observed in nocebo hyperalgesia.

## Figures and Tables

**Table 1:** Summary of characteristics of the 30 subjects.

	Introverts	Extroverts	*p* value
No.	13	17	
Age	24.54 ± 1.94	24.59 ± 3.16	*p* = 0.961
SAS	35.46 ± 5.03	34.53 ± 7.06	*p* = 0.690
SDS	32.92 ± 4.61	34.88 ± 5.24	*p* = 0.295
Vas pain status (baseline)	3.85 ± 1.07	3.59 ± 0.71	*p* = 0.434
Vas placebo intervention	2.92 ± 1.04^a^	1.94 ± 0.66^b^	*p* = 0.004
Vas nocebo intervention	5.31 ± 1.32^c^	4.18 ± 0.95^d^	*p* = 0.011

^a^Placebo status vs. pain status (introverts), ^b^placebo status vs. pain status (extroverts), ^c^nocebo status vs. pain status (introverts), and ^d^nocebo status vs. pain status (extroverts). ^a^*p* = 0.001, ^b^*p* < 0.001, ^c^*p* < 0.001, and ^d^*p* = 0.008.

**Table 2:** Locations of the regions showing significantly altered EC with the rACC in placebo status compared with pain status in IG (*p* < 0.05, FDR < 0.05).

Brain region	R/L	MNI			Voxel	*t* value
		*X*	*Y*	*Z*		
CPL	R	12	-84	-33	73	-5.712
PHP	L	-21	-36	-15	82	-8.6931
FG	L	-42	-21	-24	41	6.0333
PHP	R	15	-36	-6	37	-7.2699
IC	L	-42	15	0	69	-7.4654
THS	L	-9	-9	12	51	-6.5252
THS	R	9	-9	15	93	-5.8372
DLPFC	R	24	36	24	39	-6.0661
PCC	R	6	-30	33	39	-6.8027
SMA	L	-21	-12	57	56	7.6427

Abbreviations: FDR: false discovery rate; MNI: Montreal Neurological Institute; CPL: cerebellum posterior lobe; PHP: parahippocampal gyrus; FG: fusiform gyrus; IC: insular; THS: thalamus; DLPFC: dorsolateral prefrontal cortex; PCC: posterior cingulate cortex; SMA: supplementary motor area.

**Table 3:** Locations of the regions showing significantly altered EC with the rACC in placebo status compared with pain status in EG (*p* < 0.05, FDR < 0.05).

Brain region	R/L	MNI			Voxel	*t* value
		*X*	*Y*	*Z*		
AMYG	L	-21	-9	-15	132	-6.2909
MTL	R	57	-27	-18	489	-7.4063
CPL	L	-30	-48	-15	71	-5.7323
BRS	L	-3	-33	-12	31	5.8914
LG	R	21	-96	-15	214	-7.6591
HP	R	27	-15	-21	40	-5.6226
ITL	L	-45	-54	-12	133	-6.1497
OFC	R	39	21	-9	115	-6.2186
AG	R	42	-84	27	97	-6.4737
pgACC	R	18	30	12	904	-10.4007
S2	R	69	-27	33	47	-6.0735
SMA	L	-3	6	57	813	-10.511
DLPFC	L	-27	42	36	115	-7.6317
VMPFC	R	12	45	30	254	-7.9006
Precuneus	L	-3	-66	45	208	-7.3632

Abbreviations: FDR: false discovery rate; MNI: Montreal Neurological Institute; AMYG: amygdala; MTL: middle temporal lobe; CPL: cerebellum posterior lobe; BRS: brainstem; LG: lingual gyrus; HP: hippocampal gyrus; ITL: inferior temporal lobe; OFC: orbitofrontal cortex; AG: angular gyrus; pgACC: pregenual anterior cingulate cortex; S2: secondary somatosensory area; SMA: supplementary motor area; DLPFC: dorsolateral prefrontal cortex; VMPFC: ventromedial prefrontal cortex.

**Table 4:** Locations of the regions showing significantly altered EC with the rACC in IG compared with EG in placebo status (*p* < 0.05, FDR < 0.05).

Brain region	R/L	MNI			Voxel	*t* value
		*X*	*Y*	*Z*		
CAL	L	-27	-39	-30	42	-6.4658
HP	R	27	-18	-21	33	6.0951
RO	R	63	-3	9	113	6.2625
IC	R	18	30	9	45	6.8961
PCC	R	6	-33	9	60	5.8006
MCC	L	-3	-6	33	44	6.2217
SMA	L	-18	-12	60	44	7.3024

Abbreviations: FDR: false discovery rate; MNI: Montreal Neurological Institute; CAL: cerebellum anterior lobe; HP: hippocampal gyrus; RO: rolandic operculum; IC: insular; PCC: posterior cingulate cortex; MCC: midcingulate cortex; SMA: supplementary motor area.

**Table 5:** Locations of the regions showing significantly altered EC with the rACC in nocebo status compared with pain status in IG (*p* < 0.05, FDR < 0.05).

Brain region	R/L	MNI			Voxel	*t* value
		*X*	*Y*	*Z*		
CPL	R	30	-72	-57	82	-5.2554
BRS	R	12	-18	-9	79	8.7978
BRS	L	-15	-30	-42	41	5.9759
ITL	R	63	-6	-18	46	7.4626
OFC	L	-33	33	-12	32	7.9529
DLPFC	R	24	60	12	117	8.6465
S1	L	-36	0	33	57	6.717
DLPFC	R	30	33	39	43	6.5439

Abbreviations: FDR: false discovery rate; MNI: Montreal Neurological Institute; CPL: cerebellum posterior lobe; BRS: brainstem; ITL: inferior temporal lobe; OFC: orbitofrontal cortex; DLPFC: dorsolateral prefrontal cortex; S1: primary somatosensory area.

**Table 6:** Locations of the regions showing significantly altered EC with the rACC in nocebo status compared with pain status in EG (*p* < 0.05, FDR < 0.05).

Brain region	R/L	MNI			Voxel	*t* value
		*X*	*Y*	*Z*		
BRS	L	-3	-30	-18	36	6.7303
CAU	L	-24	9	6	37	5.5236
IC	L	-42	-12	21	70	6.3796

Abbreviations: FDR: false discovery rate; MNI: Montreal Neurological Institute; BRS: brainstem; CAU: caudate; IC: insular.

**Table 7:** Locations of the regions showing significantly altered EC with the rACC in IG compared with EG in nocebo status (*p* < 0.05, FDR < 0.05).

Brain region	R/L	MNI			Voxel	*t* value
		*X*	*Y*	*Z*		
CPL	L	-12	-78	-48	52	-6.9504
BRS	R	3	-9	-21	38	6.5114
ITL	R	66	-12	-6	46	5.6759
OFC	L	-24	27	-18	43	7.446
MTL	R	57	-45	0	42	-4.817
DLPFC	R	54	12	12	44	6.4395
IC	L	-30	-15	24	35	-6.3577

Abbreviations: FDR: false discovery rate; MNI: Montreal Neurological Institute; MTL: middle temporal lobe; CPL: cerebellum posterior lobe; BRS: brainstem; ITL: inferior temporal lobe; OFC: orbitofrontal cortex; DLPFC: dorsolateral prefrontal cortex; IC: insular.

**Table 8:** Locations of the regions showing significantly altered EC with the rACC in placebo response compared with nocebo response in IG (*p* < 0.05, FDR < 0.05).

Brain region	R/L	MNI			Voxel	*t* value
		*X*	*Y*	*Z*		
CPL	L	-6	-75	-30	63	-7.8966
CPL	R	48	-48	-33	41	-6.1458
BRS	R	9	-21	-12	40	-6.4725
FG	L	-42	-21	-24	50	5.6872
THS	R	12	-12	15	41	-6.268
PHP	L	-24	-33	-12	58	-7.0699
HP	R	6	-33	9	30	5.7866
MCC	L	-3	27	33	72	-6.3501
IC	L	-39	0	21	53	-6.032
DLPFC	R	24	36	24	127	-6.989
DLPFC	L	-18	51	27	36	-4.8449

Abbreviations: FDR: false discovery rate; MNI: Montreal Neurological Institute; CPL: cerebellum posterior lobe; BRS: brainstem; FG: fusiform gyrus; THS: thalamus; PHP: parahippocampal gyrus; HP: hippocampal gyrus; MCC: midcingulate cortex; IC: insular; DLPFC: dorsolateral prefrontal cortex.

**Table 9:** Locations of the regions showing significantly altered EC with the rACC in placebo response compared with nocebo response in EG (*p* < 0.05, FDR < 0.05).

Brain region	R/L	MNI			Voxel	*t* value
		*X*	*Y*	*Z*		
CPL	R	15	-75	-24	35	-5.1102
THS	R	9	-15	0	60	-6.5571
THS	L	-15	-21	12	478	-9.1942
RO	L	-63	3	18	176	-7.6485
CAU	R	21	-9	18	93	-6.5686
PHP	R	18	-21	-18	65	-6.535
pgACC	L	-12	24	21	36	-6.2185
MCC	L	-12	12	30	80	-6.8046
MTL	R	60	-48	3	76	-6.2333
MFL	R	36	21	51	76	-7.6418
SMA	R	15	21	51	259	-9.06

Abbreviations: FDR: false discovery rate; MNI: Montreal Neurological Institute; CPL: cerebellum posterior lobe; THS: thalamus; RO: rolandic operculum; CAU: caudate; PHP: parahippocampal gyrus; pgACC: pregenual anterior cingulate cortex; MCC: midcingulate cortex; MTL: middle temporal lobe; MFL: middle frontal lobe; SMA: supplementary motor area.

## Data Availability

The datasets used and/or analysed during the current study are available from the corresponding author on reasonable request.

## Supplementary Materials

Supplementary Materials Table S1: comparison of VAS scores before two interventions.
