# Supplementary material for: Personality Differences of Brain Networks in Placebo Analgesia and Nocebo Hyperalgesia: A Psychophysiological Interaction (PPI) Approach in fMRI
Source: Neural Plast. 2020 Oct 19;2020:8820443. doi: 10.1155/2020/8820443 (PMC7591942; doi:10.1155/2020/8820443)
Supplement: Supplementary Materials — Table S1: comparison of VAS scores before two interventions. [file 8820443.f1.docx]

Statistical analysis showed that the pain state was stable in both groups during the 20-minute washout period (see Table S1).

Table S1. Comparison of VAS scores before two interventions.

|  | Introverts | Extroverts | *p*-value |
| --- | --- | --- | --- |
| Pre-first intervention | 3.85±1.07 | 3.59±0.71 | *p=0.434* |
| Pre-Second intervention | 3.38±0.51 | 3.71±0.85 | *p=0.237* |
| *p*-value | *p=0.190* | *p=0.651* |  |
